# Case report: Atypical case of autoimmune glial fibrillary acidic protein astrocytopathy following COVID-19 vaccination refractory to immunosuppressive treatments

**DOI:** 10.3389/fimmu.2024.1361685

**Published:** 2024-04-11

**Authors:** Yuto Morishima, Takanori Hata, Sho Nakajima, Kazumasa Shindo, Mai Tsuchiya, Tsubasa Watanabe, Ippei Tahara, Tetsuo Kondo, Akio Kimura, Takayoshi Shimohata, Yuji Ueno

**Affiliations:** ^1^ Department of Neurology, University of Yamanashi, Chuo, Japan; ^2^ Department of Pathology, University of Yamanashi, Chuo, Japan; ^3^ Department of Neurology, Gifu University Graduate School of Medicine, Gifu, Japan

**Keywords:** GFAP-astrocytopathy, anti-GFAP antibody, COVID-19 vaccination, autoimmune encephalitis, immunosuppressive treatments

## Abstract

A 54-year-old Japanese man presented with headache and fever the day after SARS-CoV-2 vaccination. He became deeply unconscious within a week. Brain MRI showed periventricular linear enhancements and a few spotty lesions in the cerebral white matter. Cerebrospinal fluid (CSF) testing showed mild pleocytosis. He was treated with intravenous methylprednisolone and plasma exchange. However, the white matter lesions enlarged to involve the brainstem and cerebellum, and long cord spinal lesions appeared. Anti-glial fibrillary acidic protein (GFAP) antibody was positive in the CSF and serum, and he was therefore diagnosed as autoimmune GFAP-astrocytopathy (GFAP-A). In addition, high-dose immunoglobulin therapy was administered twice, but his symptoms did not improve; the white matter lesions enlarged further, and modified Rankin Scale score increased to 5. A brain biopsy specimen showed infiltration of macrophages and CD_4_^+^ lymphocytes together with neuron and oligodendrocytic injuries and glial scar. Although GFAP-A generally responds well to steroids, the present case developed GFAP-A following SARS-CoV-2 vaccination, with refractory to intensive immunosuppressive therapy and atypical pathologic findings of infiltration of CD_4_^+^ lymphocytes and demyelination.

## 1 Introduction

Glial fibrillary acidic protein (GFAP) is an intercellular filament protein that exists mainly in astrocytes. Autoimmune GFAP-astrocytopathy (GFAP-A) was first reported by Fang in 2016, as a central nervous system (CNS) inflammatory disease with anti-GFAP antibody in the cerebrospinal fluid (CSF) (1). It causes various neuropsychiatric symptoms similar to other central autoimmune diseases (2). Although GFAP-A shows a good response to immunosuppressive treatment, some cases have had poor prognoses (2). Factors related to refractory and severe cases of GFAP-A have not been fully studied so far.

SARS-CoV-2 vaccination has been shown to cause acute disseminated encephalomyelitis (ADEM). The symptoms are generally mild, and the disease responds well to treatment, but a fatal case has been previously reported (3).

Here we report a case of GFAP-A that developed after SARS-CoV-2 vaccination, involving disseminated encephalopathy combined with extensive longitudinal myelopathy, refractory to any immunosuppressive therapies.

## 2 Case report

A 54-year-old Japanese man, with a history of poorly managed hypertension and diabetes mellitus, was taking 1000 mg of metformin, 50 mg of sitagliptin, and 40 mg of losartan daily, and he had no diabetic complications. He developed headache and fever on the day after his 5^th^ vaccination with Pfizer/BioNTech SARS-CoV-2 mRNA vaccine. Six days after onset, he was referred to another hospital for nausea, vomiting, and high-grade fever (38.8°C). His consciousness was E3V2M5 in Glasgow Coma Scale (GCS), with mildly deteriorated. Although brain computed tomography (CT) was unremarkable, CSF examination showed mild pleocyte elevation with a cell count of 86/μL (mononuclear-dominant). Intravenous treatment with acyclovir (10 mg*/*kg every 8 hour) was started due to a suspicion of herpes encephalitis. The next day, his GCS score worsened to E1V1M2, and intratracheal intubation was performed. Brain magnetic resonance imaging (MRI) showed hyperintense lesions in the medial temporal lobe and nearby bilateral ventricles. On CSF examination, the cell count was increased to 105/μL (mononuclear-dominant, 97/μL), and he was transferred to our hospital for further examination and treatment. Since tests for herpes simplex virus and varicella zoster virus DNA were negative at the previous hospital, acyclovir administration had been stopped before the transfer.

On admission, his body temperature was 36.7°C. He was intubated but had spontaneous breathing. On neurological examination, he was comatose (GCS score E1VTM1) on no sedative agents. He showed no response to sound, light, or pain. His pupils were isocoric (3 mm each), but the light reflex was absent, and the oculocephalic reflex was absent. Hypotonia and flaccidity were observed in all four limbs. Deep tendon reflexes were absent, and Babinski and Chaddock reflexes were absent. Signs of meningeal irritation were absent.

**Figure 1:**
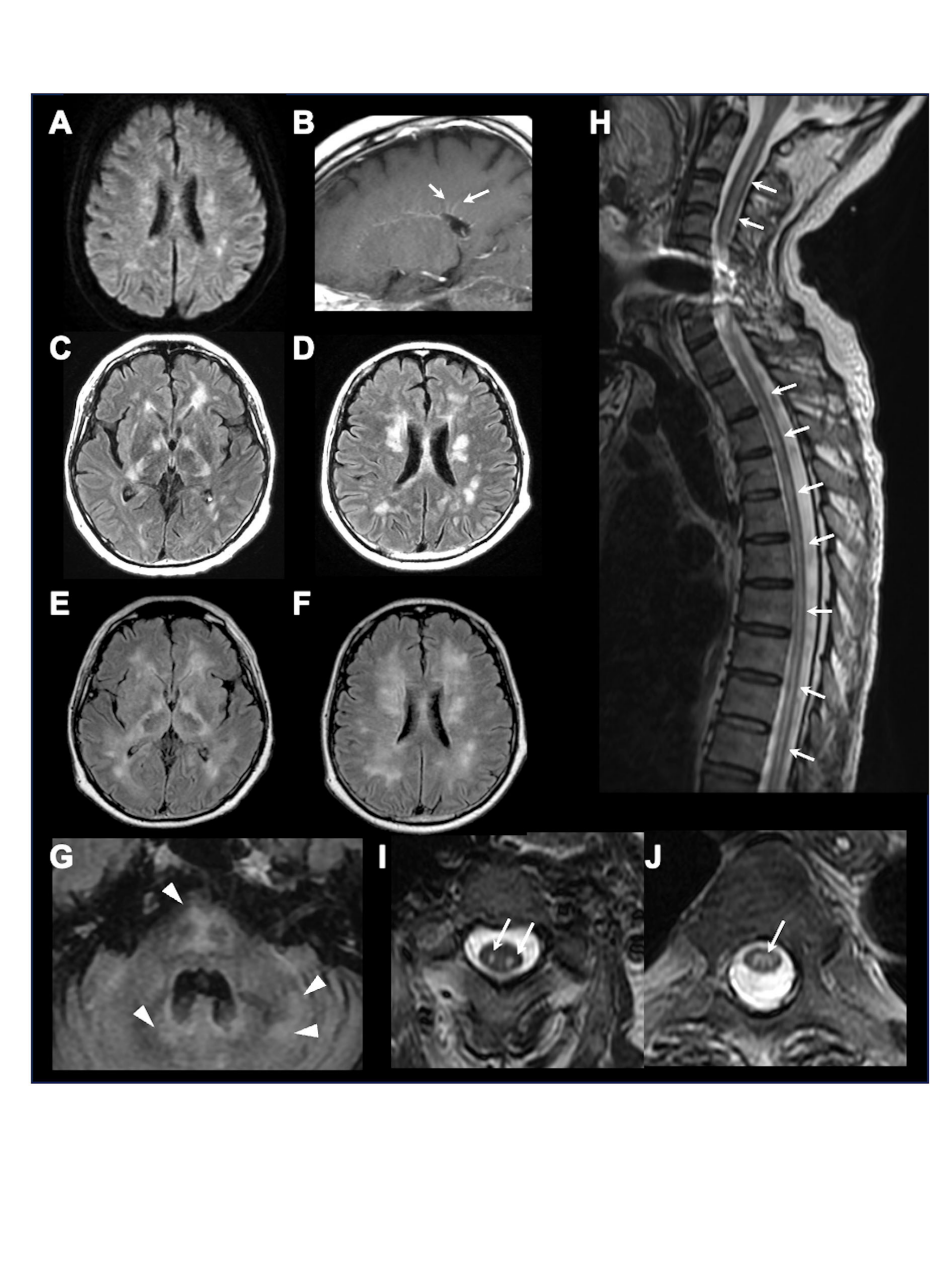
Brain and spinal MRI. Representative brain and spinal MRI in our hospital on admission, showing multiple lesions on diffusion-weighted imaging **(A)** and periventricular linear gadolinium enhancement (arrows) **(B)** on the 1^st^ day, multiple confluent lesions in the bilateral deep white matter **(C, D)** on the 15^th^ day, further enlarged lesions **(E, F)** with newly appeared lesions in the brainstem and cerebellum (arrowheads) **(G)** on the 36^th^ day, and longitudinal spinal cord lesions (arrows) on sagittal **(H)** and axial (**I**, C3 level; **J**, Th5 level) T2-weighted-imaging.

Blood tests showed mild hepatic and renal impairment, hypernatremia due to overcorrection (Na 157 mmol/L), and impaired glucose tolerance (HbA1c 7.6%, casual blood glucose 332 mg/dL). As for infections, syphilis, hepatitis B surface antigen, hepatitis C virus antibody, human T-cell leukemia virus type 1 antibody, β-D-glucan, cytomegalovirus antigenemia, quantiferon, and cryptococcus antigen were all negative (cryptococcus antigen was also negative in CSF). Antibodies such as anti-nuclear, anti-SS-A, anti-SS-B, anti-thyroglobulin, anti-thyroid peroxidase, anti-aquaporin-4, anti-voltage-gated potassium channel (leucine-rich glioma-inactivated 1 protein, contactin-associated protein-like 2), anti-myelin oligodendrocyte glycoprotein, and anti-glutamic acid decarboxylase (GAD) antibodies, proteinase3-ANCA, myeloperoxidase-ANCA, and paraneoplastic syndrome antibodies (amphiphysin, CV2, Ri, Yo, Hu, recoverin, SRY-related HMG-Box gene 1, titin, zinc-finger protein of the cerebellum 4, GAD65, Tr [Delta/Notch-like Epidermal Growth Factor-Related Receptor]) were all negative. Soluble interleukin-2 receptor was elevated to 993 U/mL. IgM- and IgG-type SARS-CoV-2 antibodies were negative, and spike IgG was positive, indicating that he acquired antibodies for COVID-19 through vaccination and not infection. CSF examination showed a cell count of 209/μL (mononuclear-dominant, 208/μL) and a glucose level of 164 mg/dL. Oligoclonal banding was negative. Anti-NMDA receptor antibody and JC virus DNA on PCR were negative. Importantly, anti-GFAP antibody was positive based on the cell-based assay (CBA) and tissue-based assay (TBA), and later serum anti-GFAP antibody was also found to be positive in CBA. CSF culture and cytology were negative. An electroencephalogram (EEG) showed a generalized slow wave, and epileptic discharges were absent. Repeated MRI showed multiple hyperintense lesions in the cerebral white matter lesions with periventricular linear enhancement (**Figures 1A, B**). Whole-body enhanced CT showed no neoplastic lesions.

**Figure 2:**
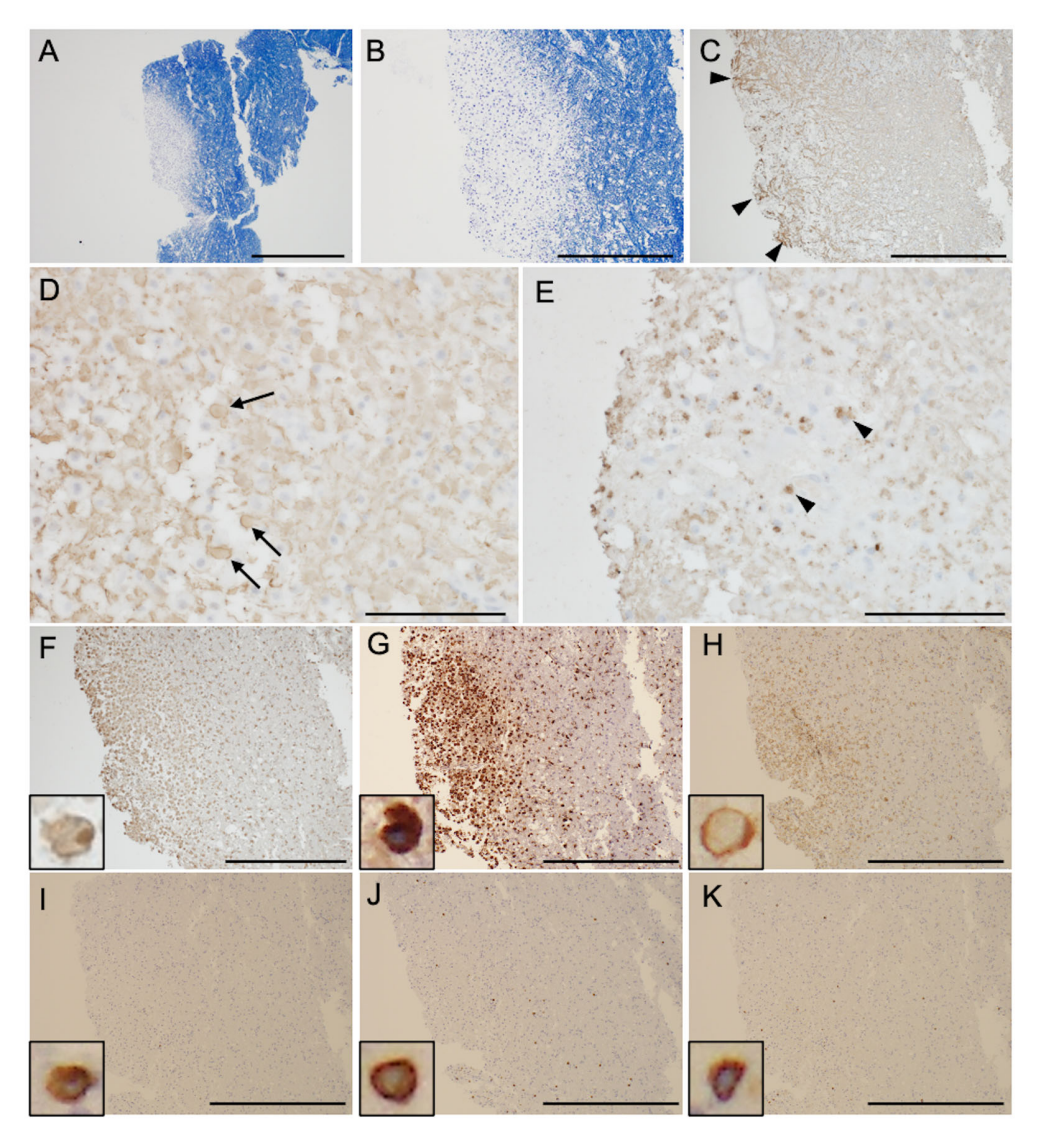
Brain biopsy samples. Klüver-Barrera staining shows that the lesion is well demarcated (**A**, magnified view in panel **B**). Glial fibrillary acid protein **(C)**, phosphorylated neurofilament heavy chain **(D)**, and myelin basic protein staining **(E)** show glial scar formation (arrowheads), spheroid neurofilaments (arrows), and decreased myelin density with phagocytosis (arrowheads), respectively. Immunohistochemical testing shows ionized calcium-binding adapter molecule-1^+^ (**F**, inset), CD_68_^+^ (**G**, inset), and CD_4_^+^ cells (**H**, inset) infiltrate into cerebral parenchyma. Infiltration of CD_3_^+^ (**I**, inset), CD_8_^+^ (**J**, inset), and CD_20_^+^ cells (**K**, inset) is scarce. Scale bar = 1 mm **(A)**, 500 μm **(B, C)**, 100 μm **(D, E)**, and 500 μm **(F–K)**.

**Figure 3:**
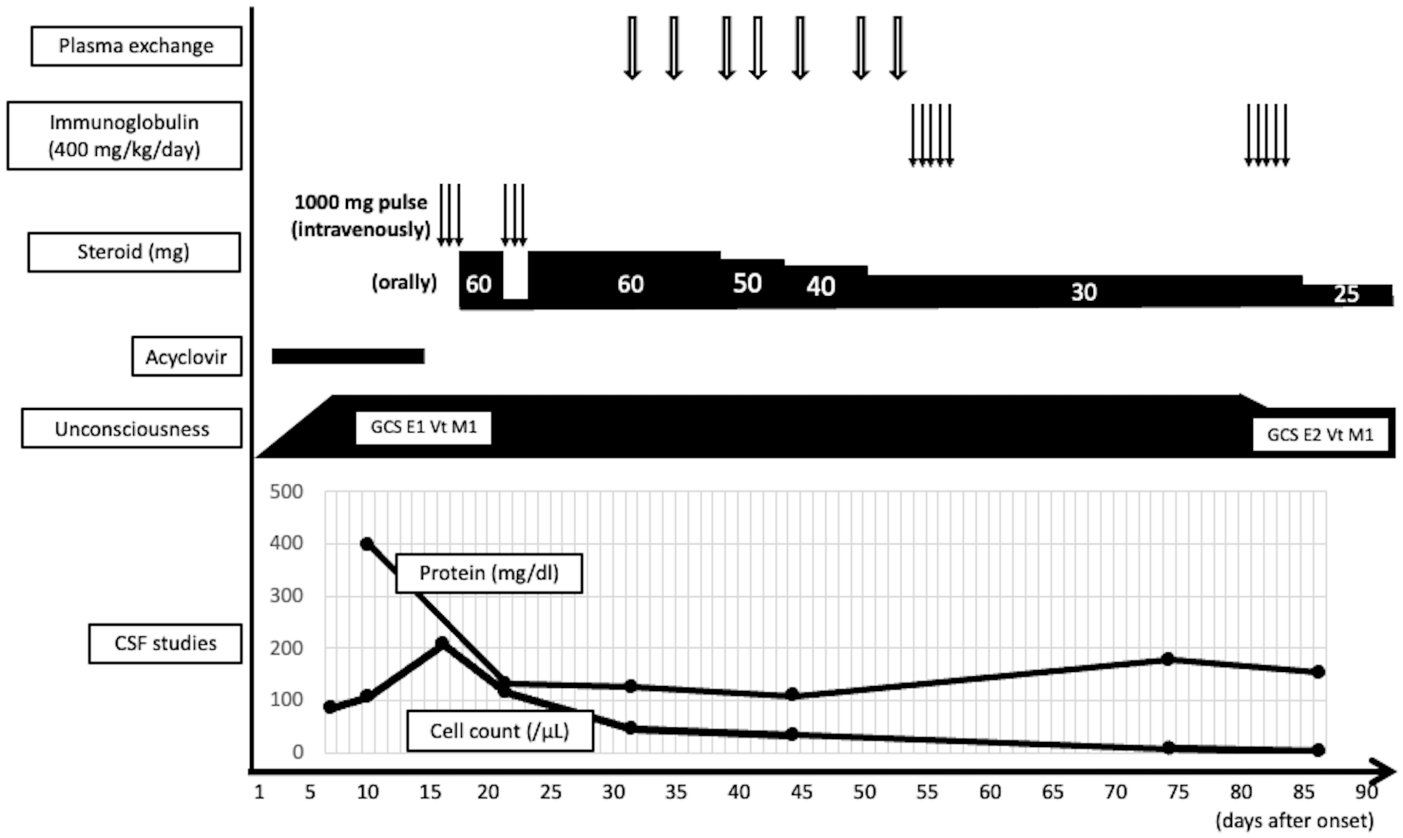
Clinical course of the present case.

Neuroimmunological disease, including GFAP-A, was suspected. From the day of transfer, we intravenously administered methylprednisolone (1000 mg/day, 3 days) twice, followed by oral prednisolone (60 mg/day). Hypernatremia was controlled to appropriate values (≒ 140 mEq/L) by fluid replacement within one week, and blood glucose was also managed by subcutaneous injection of insulin to < 200 mg/dL within two weeks. Tracheotomy was performed on the 8^th^ day of admission, and respiratory support was withdrawn, but deterioration of consciousness persisted. On the 15^th^ day of admission to our hospital, follow-up MRI showed that the lesions in the bilateral deep white matter were enlarged (**Figures 1C, D**). Plasma exchange was performed 7 times, but his comatose state did not improve, and hiccups appeared. Brain MRI on the 36^th^ day showed further enlargement of lesions, along with newly appeared lesions in the brainstem and cerebellum (**Figures 1E–G**). Spinal MRI showed extensive longitudinal spinal cord lesions (**Figure 1H–J**). Because anti-GFAP antibody in the CSF and serum was positive, he was diagnosed as GFAP-A, and intravenous immunoglobulin (400 mg/kg/day, 5 days) was administered from the 38th day, but it did not improve his consciousness level. Brain biopsy of a white matter lesion near the right frontal horn was performed on the 44^th^ day. Klüver-Barrera staining showed that the lesion was well demarcated (**Figures 2A, B**). On immunohistochemical staining, glial fibrillary acid protein, phosphorylated neurofilament heavy chain, and myelin basic protein staining showed glial scar formation, spheroid neurofilaments, and decreased myelin density with phagocytosis, respectively. (**Figures 2C–E**). As for inflammatory cells, there was marked infiltration of ionized calcium-binding adapter molecule-1^+^, CD_68_^+^, and CD_4_^+^ cells (**Figures 2F–H**), whereas CD_3_^+^, CD_8_^+^, and CD_20_^+^ cells were scarce (**Figures 2I–K**). There were no findings of lymphoma. These data indicated prominent axon and myelin injury and glial scar formation together with infiltration of macrophage and lymphocytes after intensive immunosuppressive therapies. The patient was treated with an additional high dose of immunoglobulin therapy using the same regimen. Finally, he opened his eyes with stimulation, and the oculocephalic reflex appeared soon after these therapies were administered. However, he showed no pursuit and could not communicate at all (E2VTM1 on GCS). Pleocytosis improved over time, as shown in **Figure 3**. EEG showed posterior-dominant slow alpha-waves. On the final MRI on the 77^th^ day, the cerebellum and brainstem lesions were decreased, but atrophy progressed. The cerebral lesions did not decrease, and some lesions showed low intensity on T1 and T2 weighted images on MRI, reflecting neuronal dropout. The longitudinal spinal cord lesions were not alleviated. Azathioprine (50 mg/day) was administered, and prednisolone was tapered to 15 mg/day. He was transferred to an extended-care hospital on the 141st day.

## 3 Discussion

Herein we reported a case of GFAP-A that developed one day after the 5^th^ SARS-CoV-2 vaccination and progressed to severe unconsciousness within 1 week with progressively expanding lesions in the bilateral cerebrum, brainstem, cerebellum, and spinal cord. GFAP antibody was positive in the CSF and serum. The present case had a poor response to all intensive immunosuppressive therapies, even though he lacked other noteworthy comorbidities. Pathological study indicated that macrophage and CD_4_^+^ lymphocyte infiltrations were abundant despite immunosuppressive therapy.

Since GFAP is present within glial cells, anti-GFAP antibody itself is presumably nonpathogenic. GFAP-A causes various neuropsychiatric symptoms (2), and it can also cause area postrema syndrome with a probability of 11% (4). On MRI, GFAP-A is characterized by periventricular linear enhancement. It often causes long spinal cord lesions, but the lesions are rarely confined to myelitis (5). In the present case, headache, unconsciousness, and other symptoms including hypotonia were compatible with those of GFAP-A in a previous case series (2). GFAP-A is also known to occasionally cause hiccups as a symptom of area postrema syndrome (4). MRI characteristics such as periventricular linear enhancement, cerebral multiple lesions, and long cord spinal lesions are also consistent with GFAP-A. Thus, the clinical characteristics and radiological findings in the present case were similar to those of GFAP-A except for being treatment-refractory and the patient having a poor functional prognosis.

Regarding treatment for GFAP-A, steroids, immunoglobulins, and blood purification have been effective in a previous study, with response rates of 87.5%, 75%, and 50%, respectively (2). In contrast, 17.5% of the cases in a French cohort had GFAP-A relapse, and some of them had sequelae (6). In particular, a report of seven cases with severe GFAP-A showed that most cases responded to treatment (7), but their condition worsened with disease recurrence. Therefore, GFAP-A may have a poor prognosis in the long-term. In the present case, intravenous methylprednisolone, plasma exchange, and immunoglobulin therapy were consistently ineffective, and the lesions in the brain and spinal cord enlarged. Thus, this was a rare case that was refractory to any immunosuppressive treatments and showed a much worse outcome even when compared to previously reported cases (2, 7).

GFAP-A could be present in other neuroimmunological disorders such as those that are positive for anti-NMDA receptor antibody, neoplastic diseases including teratoma, adenocarcinoma, glioma (2), diffuse large B-cell lymphoma (8), and lymphomatoid granulomatosis (9). Although the present case did not have any comorbid disorders, GFAP-A developed soon after SARS-CoV-2 vaccination. Previously, there was a case of GFAP-A that developed 4 days after the second SARS-CoV-2 vaccination (10). Although the pathology of GFAP-A is heterogeneous, many studies demonstrated that the infiltration of CD_8_^+^ lymphocytes was predominant in GFAP-A. Iorio et al. performed a leptomeningeal biopsy, which showed a necrotizing inflammatory process with infiltration of CD_8_^+^ lymphocytes and macrophages (12). A pathological study of 8 cases of GFAP-A showed marked perivascular infiltration of CD_8_^+^ lymphocytes, as well as diffuse infiltration of CD_68_^+^ and CD_163_^+^ macrophages (7). Moreover, it was shown that demyelination was uncommon in GFAP. In the present case, marked infiltration of macrophages and CD_4_^+^ lymphocytes together with neuronal and oligodendrocytic injuries and glial scar were seen after immunosuppressive therapy. Meanwhile, SARS-CoV-2 vaccination has been shown to cause ADEM, and a pathological study showed infiltration of CD_68_^+^ macrophages and lymphocytes and parenchymal hemorrhage (3, 11). Collectively, our case exhibited atypical clinical features of GFAP-A in terms of refractory to immunosuppressive treatments, and infiltration of CD_4_^+^ lymphocytes and demyelination in the pathology.

It should be noted that the serum anti-GFAP antibody was shown as positive in our case. Previously, serum anti-GFAP antibody was positive in 5% (6), 55% (2), and 100% (12) of patients with GFAP-A based on the CBA or TBA. It is suggested that intrathecal production of anti-GFAP antibody could simply leak to the blood by disruption of blood-CSF barrier due to GFAP-A. On the contrary, it is hypothesized that the serum anti-GFAP antibody could be triggered by SARS-CoV-2 vaccination, which induced the infiltration of macrophage and CD_4_^+^ lymphocytes, and resulted in demyelination and refractory to immunosuppressive therapy. So far, the pathogenic role of serum anti-GFAP antibody in GFAP-A has been unknown. Further study involving atypical GFAP-A cases is warranted.

## 4 Conclusions

GFAP-A might be induced by SARS-CoV-2 vaccination, with extensive involvement of the cerebrum, brainstem, cerebellum, and spinal cord, and be refractory to any immunosuppressive therapy. Our case was atypical GFAP-A regarding refractory to immunosuppressive treatments, and infiltration of CD_4_^+^ lymphocytes and demyelination. The pathogenesis of GFAP-A is still not fully understood, and further study is needed to identify aggravating factors and to obtain new insights for treatment in severe cases.

## Data availability statement

The original contributions presented in the study are included in the article/supplementary material. Further inquiries can be directed to the corresponding author.

## Ethics statement

The studies were conducted in accordance with the local legislation and institutional requirements. The participants provided their written informed consent to participate in this study. Written informed consent was obtained from the individual(s) for the publication of any potentially identifiable images or data included in this article.

## Author contributions

YM: Writing – original draft, Formal Analysis, Data curation, Conceptualization. TH: Writing – review & editing, Formal Analysis, Data curation. SN: Writing – review & editing, Formal Analysis, Data curation. KS: Writing – review & editing, Formal Analysis, Data curation. MT: Writing – review & editing, Formal Analysis, Data curation. TW: Writing – review & editing, Formal Analysis, Data curation. IT: Writing – review & editing, Formal Analysis, Data curation. TK: Writing – review & editing, Formal Analysis, Data curation. AK: Writing – review & editing, Formal Analysis, Data curation. TS: Writing – review & editing, Formal Analysis, Data curation. YU: Writing – review & editing, Writing – original draft, Data curation, Conceptualization.
